# Protocol to generate and characterize biofouling transformants of a model marine diatom

**DOI:** 10.1016/j.xpro.2021.100716

**Published:** 2021-08-05

**Authors:** Weiqi Fu, Bushra Dohai, Diana Charles El Assal, Sarah Daakour, Amnah Alzahmi, David R. Nelson, Ashish Jaiswal, Alexandra Mystikou, Mehar Sultana, James Weston, Kourosh Salehi-Ashtiani

**Affiliations:** 1 Department of Marine Science, Ocean College, Zhejiang University, Zhoushan, Zhejiang 316021, China; 2 Laboratory of Algal, Systems, and Synthetic Biology (LASSB), Division of Science and Math, New York University Abu Dhabi, P.O. Box 129188, Abu Dhabi, UAE; 3 Center for Systems Biology and Faculty of Industrial Engineering, Mechanical Engineering and Computer Science, School of Engineering and Natural Sciences, University of Iceland, Reykjavik 101, Iceland; 4 Center for Genomics and Systems Biology (CGSB), New York University Abu Dhabi, P.O. Box 129188, Abu Dhabi, UAE; 5 Core Technology Platforms, New York University Abu Dhabi, Abu Dhabi, UAE; 6 Department of Biology, United Arab Emirates University (UAEU), Al Ain, UAE

**Keywords:** Biotechnology and bioengineering, Microbiology, Model Organisms, RNAseq, Systems biology

## Abstract

Diatoms are a major group of microalgae that initiate biofouling by surface colonization of human-made underwater structures; however, the involved regulatory pathways remain uncharacterized. Here, we describe a protocol for identifying and validating regulatory genes involved in the morphology shift of the model diatom species *Phaeodactylum tricornutum* during surface colonization. We also provide a workflow for characterizing biofouling transformants. By using this protocol, gene targets such as *GPCR* signaling genes could be identified and manipulated to turn off diatom biofouling.

For complete information on the generation and use of this protocol, please refer to Fu et al. (2020).

## Highlights

• Protocol for obtaining biofouling transformants of diatoms
• Protocol includes characterization of biofouling transformants
• We use systems biology approaches to select and validate GPCR signaling genes

## Graphical abstract

**Figure:**
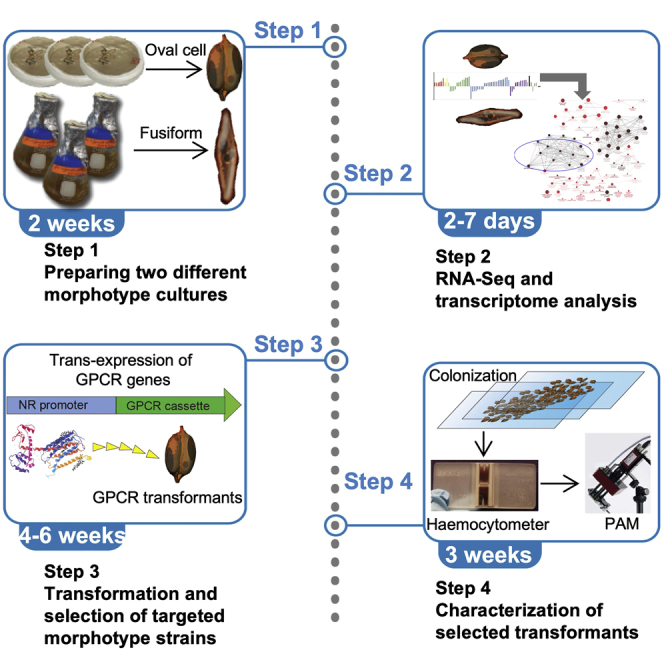

## Before you begin

This protocol was used in a recent publication (Fu et al., 2020) to generate and characterize biofouling transformants of a model marine diatom *Phaeodactylum tricornutum*. While this protocol should be thoroughly followed and may take several months to complete, there are three major steps in the whole process with three corresponding stopping points that allow the experiment to be suspended if needed.

In addition to the standard lab equipment, including safety cabinets, PCR machines, centrifuges, consumables, etc., it is critical to use best laboratory practices and workspaces to avoid contamination with RNAse and consequent mRNA degradation when working with RNA.

## Key resources table

**Table:** 

REAGENT or RESOURCE	SOURCE	IDENTIFIER
**Biological samples**		

*Phaeodactylum tricornutum*	CCAP	CCAP 1055/1
*GPCR1A* transformants of *P. tricornutum*	This paper	N/A
*GPCR4* transformants of *P. tricornutum*	This paper	N/A
Solid culture *of P. tricornutum*	This paper	N/A

**Chemicals, peptides, and recombinant proteins**		

NaNO_3_	Sigma-Aldrich or major supplier	**f/2 + Si Medium∗ (Guillard’s medium for diatoms) `**
NaH_2_PO_4_.2H_2_O	Sigma-Aldrich or major supplier	
Na_2_ EDTA	Sigma-Aldrich or major supplier	
FeCl_3_.6H_2_O	Sigma-Aldrich or major supplier	
CuSO_4_.5H_2_O	Sigma-Aldrich or major supplier	
ZnSO_4_.7H_2_O	Sigma-Aldrich or major supplier	
CoCl_2_.6H_2_O	Sigma-Aldrich or major supplier	
MnCl_2_.4H_2_O	Sigma-Aldrich or major supplier	
Na_2_MoO_4_.2H_2_O	Sigma-Aldrich or major supplier	
Cyanocobalamin (vitamin B12)	Sigma-Aldrich or major supplier	
Thiamine HCl (vitamin B1)	Sigma-Aldrich or major supplier	
Biotin	Sigma-Aldrich or major supplier	
Na_2_SiO_3_.9H_2_O	Sigma-Aldrich or major supplier	
Chelex-100	Sigma-Aldrich or major supplier	CAS No. 11139-85-8

**Critical commercial assays**		

MagMAX-96 Total RNA Isolation Kit	Thermo Fisher Scientific Inc.	Kit AM1830
KAPA Library Quantification Kit	Roche	Kapa Biosystems
DNA Clean & Concentrator^TM^-25 Kit	Zymo Research Corporation	DCC-25, Catalog No. 50444216
TruSeq V2 RNA Sample Prep Kit	Illumina	N/A

**Deposited data**		

RNA-seq data with triplicates of biological samples for the liquid culture of *Phaeodactylum tricornutum*	GenBank/NCBI database; Dryad	GenBank: PRJNA566271; Dryad with a unique identifier (https://doi.org/10.5061/dryad.ns1rn8ppx).
RNA-seq data with triplicates of biological samples for the solid culture of *P. tricornutum*	GenBank/NCBI database; Dryad	GenBank: PRJNA566271; Dryad with a unique identifier (https://doi.org/10.5061/dryad.ns1rn8ppx).
RNA-seq data with triplicates of biological samples for the liquid culture of *GPCR1A* transformants of *P. tricornutum*	GenBank/NCBI database; Dryad	GenBank: PRJNA566271; Dryad with a unique identifier (https://doi.org/10.5061/dryad.ns1rn8ppx).

**Experimental models: Organisms/strains**		

*Phaeodactylum tricornutum*	CCAP	CCAP 1055/1

**Oligonucleotides**		

Primers (forward, 5′- CACAAA CCGAACAGCCCTAC-3’; reverse, 5′- TCGAGCTTCACAACCTGTCC-3′)	Integrated DNA Technologies	Primer of GPCR1A
Primers (forward, 5′-CACAAACC GAACAGCCCTAC-3’; reverse, 5′- CAGTGACGTTGCG ACAATCC-3′)	Integrated DNA Technologies	Primer of GPCR4

**Recombinant DNA**		

pPha-NR	NCBI	GenBank: JN180663.1
Synthetic genes (DNA constructs) cloned into the vector pPha-NR	Twist Bioscience	Gene names of *Phaeodactylum tricornutum*: Phatr3_J22677, Phatr3_J41807, Phatr3_Jdraft1756, Phatr3_J10677, Phatr3_J54411, Phatr3_EG02512, Phatr3_J51511, Phatr3_J12877, Phatr3_J2097, Phatr3_Jdraft1000, Phatr3_J54505, Phatr3_J55230, Phatr3_Jdraft1740, Phatr3_J44133

**Software and algorithms**		

BLAST (Basic Local Alignment Search Tool) command-line applications developed at the National Center for Biotechnology Information (NCBI)	NCBI	N/A
BLAST2GO command line	blast2go_cli_v1.3.3	N/A
KEGG Automatic Annotation Server	KEGG: Kyoto Encyclopedia of Genes and Genomes	N/A
Cytoscape	Website: http:// www.cytoscape.org	v.3.6.0
CLUGO: a clustering algorithm for automated functional annotations based on gene ontology	Cytoscape	v2.5.0
DESeq2	R studio	Bioconductor version: Release (3.11)
STRING database	Website: https://string-db.org	N/A

**Other**		

KOD hot-start DNA polymerase	Novagen	Manufacturer Part Number: 710864
The genome of *Phaeodactylum tricornutum*	Genome assembly: ASM15095v2	blastx: 2.6.0+
AMPK signaling pathway	KEGG	https://www.genome.jp/dbget-bin/www_bget?map04152
cAMP signaling pathway	KEGG	https://www.genome.jp/dbget-bin/www_bget?map04024
FoxO signaling pathway	KEGG	https://www.genome.jp/dbget-bin/www_bget?map04068
MAPK signaling pathway	KEGG	https://www.genome.jp/dbget-bin/www_bget?map04010
mTOR signaling pathway	KEGG	https://www.genome.jp/dbget-bin/www_bget?map04150
cAMP signaling pathway	KEGG	https://www.kegg.jp/dbget-bin/www_bget?map04024
Fluorescent lights	IKEA	N/A
Spectrophotometer	Synergy H1	N/A
Laminar Flow Cabinet	NuAire	N/A
Autoclave	Prestige	N/A
pH meter	Mettler Toledo	N/A
PHYTO-PAM-II	Heinz Walz GmbH	N/A
Super Electroporator Type II	NEPA21	N/A
QIAGEN Plasmid Mini Kit	QIAGEN	N/A
Illumina MiSeq and HiSeq 2500 system	Illumina	N/A
Light microscope	Olympus BX53	N/A
Hemocytometer	Nexcelom Bioscience Western Europe	N/A
Plant growth chamber	CLF Plant Climatics GmbH	N/A
Glass microscope slides	Thermo Fisher Scientific Inc.	N/A
Bioanalyser	Agilent Technologies	Agilent 2100

## Materials and equipment

For efficient extraction of total RNAs from diatom samples, plastic applicator sticks (with a length of appropriately 14 cm), which fit the 1.5 mL Eppendorf microtubes, are used to grind and break down frozen cell pellets into powders.

## Step-by-step method details

### Preparing and cultivating two different morphotype cultures

**Timing:** 2 **weeks**

1. Grow diatoms on solid plates
  a. Make up a fresh medium according to the f/2+Si medium recipe (Table 1).

**Table 1:** Recipe of f/2 +Si medium^∗^

Components	Final concentration
NaNO_3_	75 mg/L
NaH_2_PO_4_·H_2_O	5 mg/L
Na_2_SiO_3_·9H_2_O	30 mg/L
Na_2_·EDTA	4.36 mg/L
FeCl_3_·6H_2_O	3.15 mg/L
CuSO_4_·5H_2_O	0.01 mg/L
ZnSO_4_·7H_2_O	0.022 mg/L
CoCl_2_·6H_2_O	0.01 mg/L
MnCl_2_·4H_2_O	0.18 mg/L
Na_2_MoO_4_·_2_H_2_O	0.006 mg/L
Thiamine hydrochloride	0.1 mg/L
Biotin	0.5 μg/L
Vitamin B_12_	0.5 μg/L

^∗^Medium prepared using filtered natural seawater at pH=8.0. For solid plates, agar (1.2%, w/v) will be added to the liquid medium before autoclave.
  b. Autoclave for 25 min (at 121°C with a pressure of 15 psi or 1.03 × 10^5^ Pa) and cool the medium-agar mix to 55°C.
  c. Add any antibiotics if needed (for example, to prepare agar plates with 100 μg/mL zeocin for growing zeocin-resistant diatom transformants) and pour the plates with 20 mL for each plate in a 100 mm diameter plate, and then cool down the plates to room temperature (22 ± 2°C) and dry the plates in the safety cabinet (laminar flow hood) for one hour after the agar solidifies.
  d. Subculture diatoms on the freshly prepared solid plates by distributing 200–400 μL liquid seed cultures, which are cultivated under continuous cool white fluorescent lights of 50 μmol photons m^−2^ s^−1^ at 22 ± 2°C and maintained in exponential growth phase on each plate.
  e. Grow the solid cultures under continuous cool white fluorescent lights of 50 μmol photons m^−2^ s^−1^ at 22 ± 2°C until observing single colonies and oval cells as the major morphotype. Freshly prepared samples should be used for cell observation to avoid morphotype changes during storage.
2. Prepare liquid diatom cultures
  a. Make up a fresh medium according to the f/2+Si medium recipe (Table 1).
  b. Autoclave for 25 min, cool the medium to room temperature (22 ± 2°C), and add any needed antibiotics.
  c. Subculture diatoms by inoculating seed cultures into the freshly prepared liquid medium (10%–20%, v/v) in 250 mL flasks with a total culture volume of 50 mL.
  d. Grow the liquid cultures under continuous cool white fluorescent lights of 50 μmol photons m^−2^ s^−1^ at 22 ± 2°C to the exponential growth phase and confirm fusiform cells as the major morphotype in the liquid cultures. Freshly prepared samples should be used for cell observation to avoid morphotype changes during storage.

### Transcriptome analysis of two different morphotype cultures

**Timing: hours to days**

High-quality RNA should be extracted from cells for both solid culture (oval > 75%) and liquid culture (fusiform > 90%) to facilitate the downstream RNA sequencing (RNA-seq) and subsequent transcriptome analysis.

**Figure 1:**
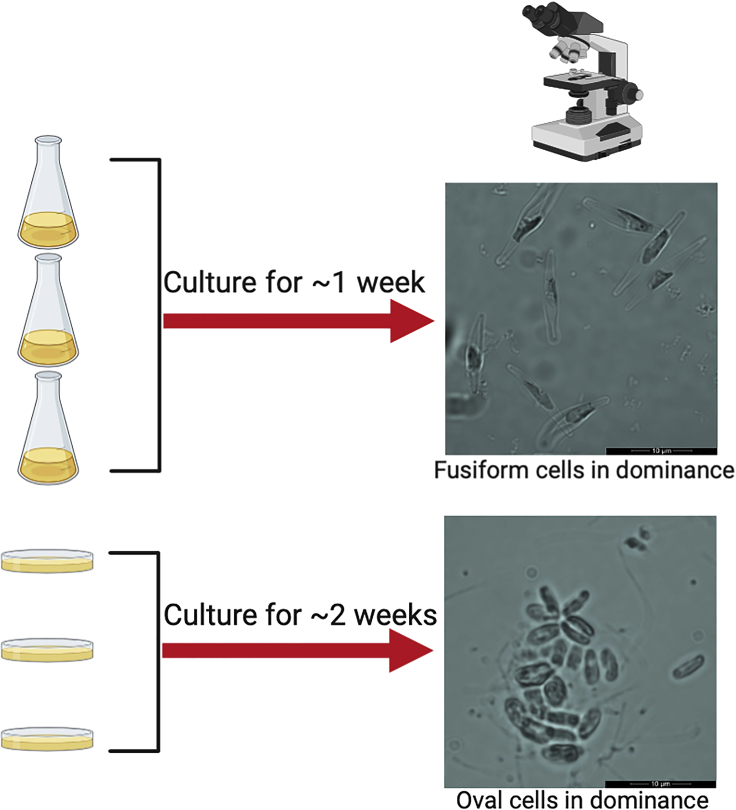
A workflow to generate liquid culture (fusiform cells in dominance) and solid culture (oval cell in dominance) There are usually three morphotypes in *P. tricornutum* cultures, i.e., fusiform, oval and triradiate cells. The fusiform cells are dominant in liquid culture under non-stress conditions, while the oval cells dominate surface colonization in solid culture. The microscopic images are representative to indicate the morphotype difference between samples.
  b. Pellet cells in 50 mL tube by centrifugation at 4000 × *g* at 20°C for 5 min, use the pipette to remove the supernatant and wash the cell pellets using deionized water; repeat the step for 2–3 times, and then resuspend cell pellets in 1 mL of deionized water.
  c. Transfer the resuspended cells to a centrifugation tube, pellet cells by centrifugation at 10000 × *g* at 20°C for 1 min and use the pipette to remove the supernatant.
  d. Freeze cell pellets in liquid nitrogen or dry ice and grind the cell pellets in 1.5 mL microtube into powder with a clean plastic applicator stick by repeating the freeze and thaw cycle five to ten times.
  e. Extract total RNA by using the MagMAX-96 Total RNA Isolation kit (AM1830, Thermo Fisher Scientific Inc.) with all related reagents according to the manufacturer’s instruction, including the steps of lysis and binding, initial nucleic acid purification, DNase treatment, and final RNA cleanup.
  f. Assess the quality of the extracted RNA samples using a Bioanalyser (Agilent 2100; Agilent Technologies, Santa Clara, CA, USA) to measure RNA Integrity Numbers (RIN). Quantify RNA samples with specific concentration, purity, and integrity values by using the Qubit RNA assay kit. A minimum RIN score of 8 is preferred for proceeding to RNA-seq.

3. Extract RNA from two different morphotype cultures for RNA-seq
  a. Collect diatom samples from fresh cultures. For both solid culture with oval cells > 75% of the population, collect cells from plates and resuspend them in deionized water; for liquid culture with fusiform cell > 90% of the population, take 30–50 mL cultures for each sample (approximately 10–20 mg dry weight for next steps 1b–1d). (Figure 1)

**CRITICAL:** The diatom cells must be ground thoroughly to break down cell walls by repeating the freeze and thaw cycle and also kept at low temperature (ice bath) conditions during the whole process to avoid any RNA degradation. In addition, the Lysis/Binding Solution for processing diatom samples must be freshly prepared and used on the same day.

4. Perform RNA-seq on RNA isolated from the WT *P. tricornutum* strain in liquid and on solid media
  a. Take the samples of total RNA with high quality after quality control using a bioanalyzer (Agilent 2100; Agilent Technologies, Santa Clara, CA, USA). The use of two or more technical replicates is recommended for each biological sample.
  b. Prepare mRNA library using Illumina TruSeq V2 RNA sample Prep Kit (San Diego, CA) according to the manufacturer’s protocol (https://www.illumina.com/products/by-type/sequencing-kits/library-prep-kits/truseq-rna-v2.html).
  c. Purify polyadenylate-containing mRNA by oligo(dT) magnetic beads from 1.0 μg of total RNA sample, fragment it, and reversely transcribe the first-strand cDNA using random primers.
  d. Then use the first-strand cDNA to synthesize second-strand cDNA by using DNA polymerase I and ribonuclease H. Treat the fragments after second-strand cDNA synthesis with end-repair, the addition of a single A base, and TruSeq adapter-index ligation to cDNA libraries.
  e. Amplify the fragment samples by PCR for 12 cycles to produce fragments with a size of 350–400 bp, including adapters, to create the final cDNA library for the next step 2f.
  f. Confirm fragment sizes and purity of the libraries with positive control by using a Bioanalyzer 2100 (Agilent 2100; Agilent Technologies, Santa Clara, CA, USA).
  g. Determine the quantities of the libraries required for RNA-seq by real-time qPCR using a KAPA library quantification kit (Kapa Biosystems, Roche) for the Illumina platform.
  h. Sequence the enriched cDNA libraries using the Miseq (Illumina) or HiSeq 2500 system (Illumina) platform with 2 × 150–base pair read length.
  i. Process data using the standard Illumina processing pipeline to segregate each multiplexed sample’s reads.
  j. Take the RNA-seq data for further analysis and deposit the raw data to the National Center for Biotechnology Information (NCBI) database.

### Preparing materials and reagents for diatom transformation

**Timing:** 3 **weeks to months**

5. Identify a group of 61 signaling genes based on a total of 2,468 up-regulated genes in the solid wild type when compared with the liquid wild type cells using the KEGG Automatic Annotation Server (http://www.genome.jp/tools/kaas/) from 44 potential signaling pathways based on KEGG database with gene annotations of the model diatom *P. tricornutum.*
6. Select 14 cDNAs encoding candidate signaling genes including several GPCR signaling genes from the 61 signaling genes with a focus on GPCR signaling genes as GPCRs are on the top level of signal transduction hierarchy.
7. Send the design request of the nucleotide sequence to the company to synthesize and clone these signaling genes in the shuttle vector pPha-NR with a nitrate reductase promoter.
8. Receive the host *E. coli* strains with the designed plasmids and extract the plasmids for diatom transformation experiments.

### Transformation and selection of targeted morphotype strains

**Timing:** 2–4 **weeks**

For the transformation of different gene constructs to the diatom *P. tricornutum*, the electroporation protocol (Miyahara et al., 2013) was used with slight modifications for ease of implementation and optimization. A NEPA21 electroporator (Nepa Gene Co. Ltd.) was used for the electroporation experiment.

9. Transform different gene constructs to the diatom *P. tricornutum*
  a. Collect diatom samples from the fresh liquid culture at the exponential growth phase (OD_600_=0.3–0.5). Take 20–30 mL cultures for each sample.
  b. Pellet cells in 50 mL tube by centrifugation at 700 × *g* at 20°C for 4 min, use the pipette to remove the supernatant and wash the cell pellets using 0.77 M mannitol; repeat the step for 2–3 times, and then resuspend cell pellets in 1 mL of 0.77 M mannitol.
  c. Linearize 3–5 μg of each plasmid construct by *Nde*I for 4 h at 37°C in a total reaction volume of 50 μL.
  d. Use DNA Clean & Concentrator^TM^-25 to recover linearized plasmids by following the manufacturer’s instructions.
  e. Mix 3–5 μg of linearized plasmids with an aliquot of 0.15 mL cell suspension on ice and then transfer the mixture to an electroporation cuvette with a 0.2 cm gap on ice.
  f. Determine the impedance of the mixture in the electroporation cuvette and adjust its impedance by using 0.77 M mannitol solution to a value between 0.4 and 0.6 kΩ.
  g. Set the electroporator and do the transformation with the following parameters: square electric poring pulses at 300V (pulse duration, 5 ms; 8 pulses; interval 50 ms; 10% decay rate), and transferring pulses at 8V (pulse duration, 50 ms; 5 pulses; interval 50 ms; 40% decay rate). Place the cuvettes with cell suspensions on ice immediately.
  h. Transfer the cell solution from ice bath within 5 min into 4 mL of f/2+Si medium in a 15 mL tube and incubate the sample at 20°C overnight or for 16–20 h under a photon flux density of 30 μmol photons m^−2^ s^−1^.
  i. Pellet cells by centrifugation at 700 × *g* at 20°C for 4 min, use the pipette to remove the supernatant and resuspend the cell pellets using 0.2 mL of f/2+Si medium.
  j. Cultivate the transformed cells on f/2+Si agar plates containing 1.2% agar and 100 μg/mL of zeocin for antibiotic resistance selection under a photon flux density of 30 μmol photons m^−2^ s^−1^. For each transformation, a minimum of two replicate plates is used.

**CRITICAL:** The diatom cells must be collected from the exponential growth phase for highly efficient transformation. Moreover, the impedance of mixed cells and plasmid DNA solution in the cuvette for electroporation must be adjusted to get a tradeoff between cell damage and DNA delivery efficiency.

10. Select successful transformants with targeted morphotypes
  a. Check the presence of single colonies on the selective agar plates after ten days to 2 weeks.
  b. Pick up single colonies and subculture them into liquid selective medium, respectively, for 1–2 weeks.
  c. Pellet cells from 1–2 mL liquid cultures by centrifugation at 10000 × *g* for 1 min, use the pipette to remove the supernatant and wash the cell pellets using deionized water; repeat the step two times, and then resuspend cell pellets in 50 μL 5% Chelex-100.
  d. Vortex for 10 s and incubate at 98°C–100°C for 10 min.
  e. Cool down on the ice for 1 min and vortex for 10 s.
  f. Spin at 10000 × *g* for 1 min and use 1 μL of supernatant for each of 20 μL PCR reaction (Table 2).

**Table 2:** Conditions for amplification PCR

Steps	Temperature	Time	Cycles
Polymerase activation	95°C	2 min	1
Denaturation	95°C	20 s	35
Annealing	59°C	10 s	35
Extension	70°C	15 s	35
Final Extension	70°C	10 min	1
Hold	4°C–8°C	forever	1
  g. Screen and verify the incorporation of plasmid DNA into the genome by checking the PCR products with corresponding primers (primers of GPCR1A and GPCR4, as indicated in the Key Resource Table).
  h. Check the morphotypes of selected transformants under light microscopy and confirm the successful transformants are with targeted morphotypes. (Figure 2)

**Figure 2:**
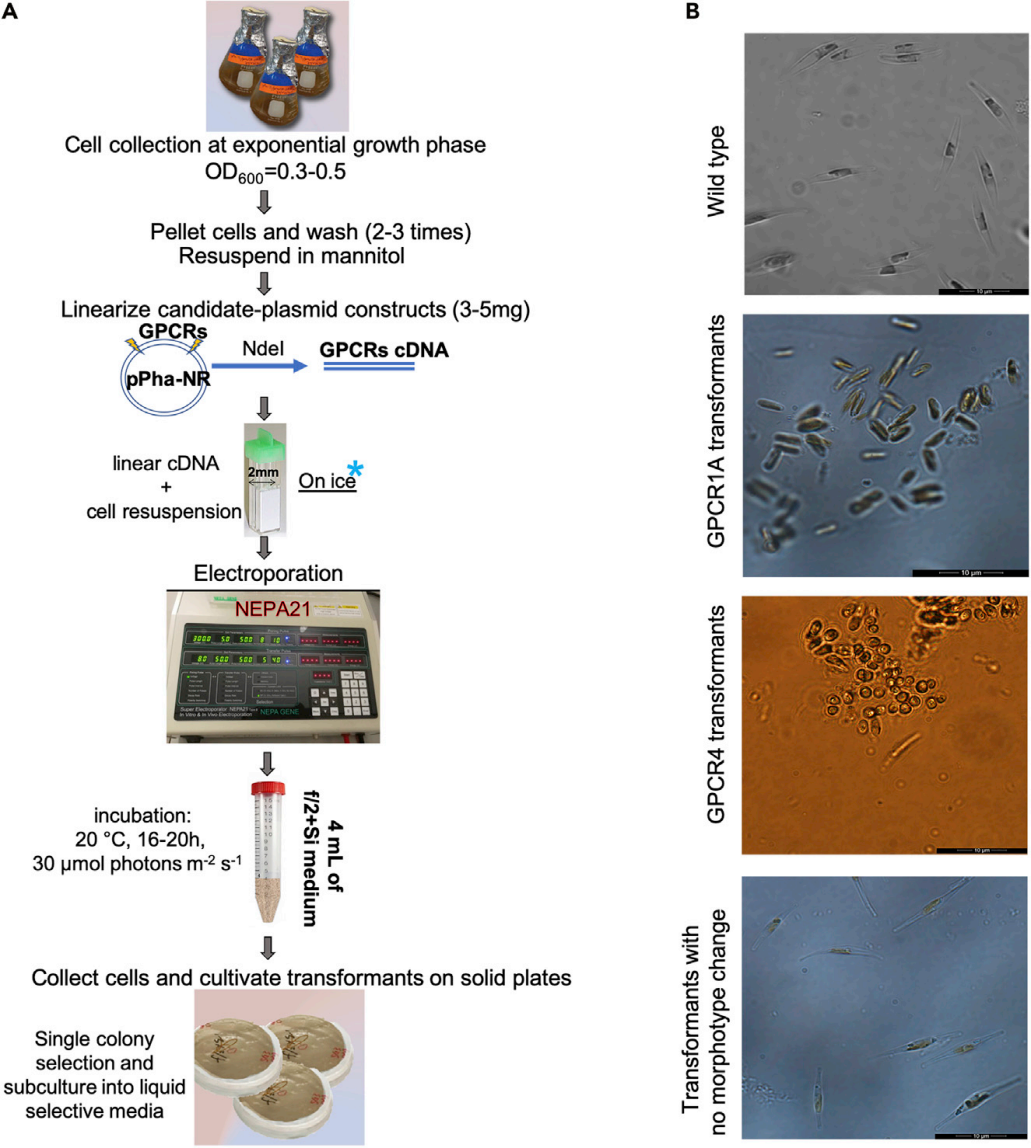
A workflow to create transformants of *P. tricornutum* (A) The major steps involved in using the electroporation approach to create transformants. (B) The microscopic images of different strains with different morphotypes, i.e., the wild type, GPCR1A transformants, GPCR4 transformants, and transformants with no morphotype change. G-protein coupled receptors (GPCRs), also known as seven-transmembrane domain receptors, mediate vital signaling processes and physiological functions in most living organisms (Shaw et al., 2019). The microscopic images are representative to indicate the morphotype difference among samples.

### Preparing samples for characterization of selected transformants

**Timing: days to weeks**

11. Prepare diatom samples for light microscopy and scanning electron microscopy (SEM) experiments
  a. Cultivate and maintain seed cultures of both the wild type and the transformants under continuous cool white fluorescent lights of 50 μmol photons m^−2^ s^−1^ at 22 ± 2°C.
  b. Subculture diatoms by inoculating seed cultures into the freshly prepared liquid medium (10%–20%, v/v) in 250 mL flasks with a total culture volume of 50 mL.
  c. Grow the liquid cultures under continuous cool white fluorescent lights of 50 μmol photons m^−2^ s^−1^ at 22 ± 2°C to exponential growth phase (optical density OD_600_ at 600 nm is between 0.1 and 0.2 in flasks) and confirm fusiform cells as the major morphotype in the wild type and oval cells as the major morphotype in the transformant liquid cultures.
  d. For the light microscopy experiment, dilute the samples to the desired cell density (between 10^5^ and 10^6^ cells/mL) and make a glass slide for microscopy observation directly; for the SEM experiment, dilute the samples to the desired cell density (between 10^5^ and 10^6^ cells/mL) for SEM observation and then remove salts by washing the samples with deionized water.
12. Prepare diatom samples for light stress, UV stress, and surface colonization experiments
  a. Cultivate and maintain seed cultures of both the wild type and the transformants under continuous cool white fluorescent lights of 50 μmol photons m^−2^ s^−1^ at 22 ± 2°C.
  b. Subculture diatoms by inoculating seed cultures into the freshly prepared liquid medium (10%–20%, v/v) in 250 mL flasks with a total culture volume of 50 mL.
  c. Grow the liquid cultures under continuous cool white fluorescent lights of 50 μmol photons m^−2^ s^−1^ at 22 ± 2°C to exponential growth phase and confirm fusiform cells as the major morphotype in the wild type and oval cells as the major morphotype in the transformant liquid cultures.
  d. Dilute the samples to the desired cell density (between 1×10^5^ and 5×10^5^ cells/mL) for each of these experiments. After dilution, samples need to be kept in the dark for one hour for the light stress experiment only.

### Characterization of selected transformants

**Timing:** 4 **days**

The morphotypes of selected transformants are determined with SEM imaging. Moreover, the transformants are characterized by the capability to respond to light stress, UV stress, and surface colonization compared to the wild type as a control.

13. SEM imaging
  a. Transfer the freshly diluted (using deionized water) samples (1–1.5 mL for each) from the previous preparation step (step 1 in the section of preparing samples for characterization of selected transformants) to microcentrifugation tubes.
  b. Dehydrate the samples by centrifuging at 5000 × *g* for 1 min, removing the supernatant with a pipette, and washing the cell pellets using 20%, 40%, 60%, 80%, and 100% ethanol gradually. A centrifugation step at 5000 × *g* for 1 min is used between washes.
  c. Vortex the cell samples in 100% ethanol.
  d. Deposit liquid solutions on Whatman track-etched membranes (VWR 514-0049).
  e. Dry the membranes and then sputter deposit approximately 2 nm of Au onto the membranes in a vacuum coater.
  f. Perform SEM imaging using a Thermo/FEI Quanta 450 Scanning Electron Microscope (or equivalent) with 5 kV accelerating voltage and a spot size (beam current) of 2 in high vacuum using an ET (Everhart-Thornley) secondary electron detector.
  g. Observe high-resolution images of individual diatom cells using SEM.

**CRITICAL:** The diatom cells must be dehydrated gradually to keep the natural structure of cells.

**Figure 3:**
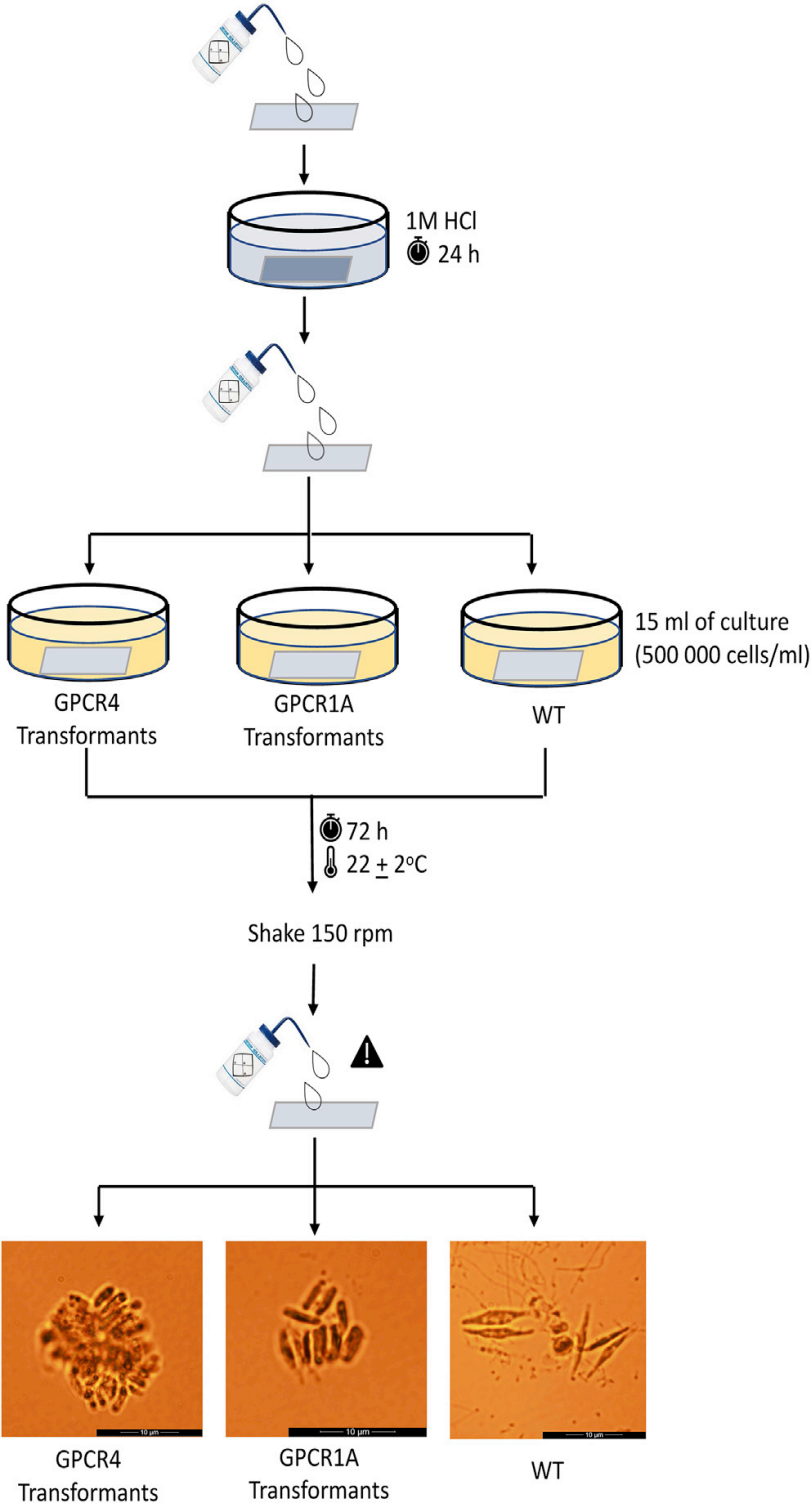
An overview of surface colonization process on glass slides The microscopic images show the morphotype difference among three different strains: the wild type, GPCR1A transformants, and GPCR4 transformants. The microscopic images are representative to indicate the morphotype difference among samples.

14. Light stress experiment
  a. Take the freshly prepared samples from the previous preparation step (step 2 in the section of preparing samples for characterization of selected transformants).
  b. Dilute the samples using f/2 + Si medium to a cell density of approximately OD_600_=0.05
  c. Keep the samples in the dark for 1 h and vortex for 10 s after dark incubation.
  d. Set the PHYTO-PAM-II (Heinz Walz GmbH) instrument with constant white light stress of 220 μmol photons m^−2^ s^−1^ for 10–15 min.
  e. Determine the effective quantum yields under different light intensities of 0, 8, 16, 58, 74, 99, 147, 236, 415, and 588 μmol photons m^−2^ s^−1^ in a consecutive mode.
  f. Compare the difference between the transformants and the wild type in terms of quantum yields.
15. UV stress experiment
  a. Take the freshly prepared samples from the previous preparation step (step 2 in the section of preparing samples for characterization of selected transformants).
  b. Dilute the samples to a cell density of 1–3 × 10^6^ cells/mL (appropriately at OD_600_ of 0.1 to 0.2) using f/2 + Si medium and transfer 3 mL of each sample to a 100 mm diameter plate
  c. Expose the samples in plates to UV light using a UV crosslinker with a UV exposure time of 60 s and energy of 0.1 J/cm^2^
  d. Incubate samples under a low light intensity of 30 μmol photons m^−2^ s^−1^ at room temperature (22 ± 2°C) for over two days.
  e. Mix the samples thoroughly by pipetting the solution and count cells to get cell density after UV treatment.
  f. Calculate the survival rate by using the initial cell density and the cell density after UV treatment and compare the difference between the transformants and the wild type
16. Surface colonization to determine the adhesive difference (Stanley and Callow, 2007) between the transformants and the wild type (Figure 3)
  a. Rinse glass microscope slides with deionized water, then soak the slides in 1M HCl for 24 h.
  b. Rinse the slides using deionized water and dry the slides.
  c. Take the freshly prepared samples from the previous preparation step (step 2 in the section of preparing samples for characterization of selected transformants) and dilute the samples to a cell density of 500,000 cells/mL (approximately at OD_600_ of 0.03).
  d. Place each slide to a 100 mm diameter dish plate.
  e. Add 15 mL of diluted samples to each dish plate.
  f. Incubate the samples for 72 h at 22 ± 2°C in growth chambers.
  g. Shake and rinse the slides gently to remove unattached cells on a shaker at 150 rpm.
  h. Count cells according to microscope images from 9 randomly sampled views.
  i. Compare the adhesive difference between the transformants and the wild type.

## Expected outcomes

The transcriptome analysis protocol will identify differentially expressed genes (DEGs) in the solid WT compared with liquid WT cells and the identification of overrepresented Gene Ontology (GO) terms for the DEGs from WT. The analysis will also allow us to identify the up-regulated genes involved in potential signaling pathways and predict their protein-protein interaction network. The ultimate expected outcome from the transcriptome analysis is to identify candidate GPCR signaling genes for transformation.

The transformation protocol will result in GPCR transformants. The selection of the transformants expressing the GPCR genes will help us sort and select for transformants that can shift cell morphotype from fusiform to oval forms when observed and compared to WT, as shown in Figure 2.

The characterization of the selected GPCR transformants will show whether they can resist light and UV stress compared to WT. The surface colonization protocol will determine these transformants' capability to attach and colonize to the surface of glass slides compared to WT, as shown in Figure 3.

## Quantification and statistical analysis

For the section of Transcriptome analysis of two different morphotype cultures, the CLC Genomics Workbench 11.0.1 (https://digitalinsights.qiagen.com) calculates the normalized Reads Per Kilobase per Million (RPKM) of genes expressed in the samples. The tool for obtaining the RPKM reads, RNA-seq Analysis, requires importing the fasta files of the sample-sequenced transcripts. A summary of the major steps is as follows:

• Import the fasta files using the “Import tracks” tool. Make sure that “Batch mode” is enabled when selecting the files to be imported.
• To run RNA-seq Analysis, go to Toolbox, Transcriptomic Analysis, then RNA-seq Analysis. Then there is a dialog box with steps to follow.
• Select the sequencing files imported previously and the reference annotation, *P. tricornutum* (2013-07-EBI-Phatr3), ASM15095v2, downloaded from EnsemblProtists ( http://protists.ensembl.org/index.html).

The detailed steps are in the manual of the CLC genomics (http://resources.qiagenbioinformatics.com/manuals/clcgenomicsworkbench). Using CLC genomics, it is optional to process RNAseq data of different samples to obtain differentially expressed genes (DEGs). Alternatively, you can use the DESeq2 R package (Love et al., 2014) for obtaining the DEGs.

To identify cellular functions enriched in the DEGs, use ClueGo plug-in (V2.5.0) (Bindea et al., 2009) in Cytoscape v3.6.0 ( https://cytoscape.org/). To do this:

• In a Cytoscape session, install the ClueGO plug-in from App menubar. Select ClueGo App to start functional analysis and Gene Set Enrichment Analysis (GSEA) of the DEGs.
• In the control panel window, select the organism, *P. tricornutum,* and paste the list of the DEGs identifiers.
• Select the visual style, *significance*, the ontologies type, *biological processes*, and enable “show only pathways with p value less than or equal to 0.05”.
• Click “*Start*” to start the analysis. ClueGO determines the statistically significant Gene Ontology (GO) terms that are overrepresented in the DEGs. ClueGO calculates the p values using a two-sided hypergeometric test and corrects them using the Benjamini-Hochberg false discovery rate (FDR).

When ClueGO finishes the analysis, the network pops up, visualizing the enriched GO terms represented in nodes. Each biological process has a specific node-color. The node size is proportional to the number of genes in a particular GO term.

• Photosynthetic efficiency

The WT and transformants’ photosynthetic performance can be evaluated using the chlorophyll fluorometer PHYTO-PAM-II (Heinz Walz GmbH). The effective quantum yields (QY) of photosystem II (PSII) is used to assess the capability of the strain to respond to immediate white light stress (220 μmol photons m^−2^ s^−1^ over 10 min). The chlorophyll fluorometer uses equation (1) to calculate the maximum photosynthetic efficiency of PSII (Table 3), where F_0_ is the ground fluorescence in the dark- or low-light adapted cells and F_m_ represents the maximal fluorescence.

**Table 3:** Example of effective PSII quantum yields measured with a chlorophyll fluorometer (PAM)

Light intensity	WT liquid sample 1	WT liquid sample 2	WT liquid sample 3	Average	Standard error
0	0.462	0.487	0.532	0.49	0.02
8	0.499	0.503	0.517	0.51	0.01
16	0.476	0.474	0.485	0.48	0.00
58	0.346	0.341	0.36	0.35	0.01
74	0.313	0.31	0.343	0.32	0.01
99	0.26	0.257	0.298	0.27	0.01
147	0.197	0.192	0.23	0.21	0.01
236	0.171	0.127	0.155	0.15	0.01
415	0.078	0.075	0.094	0.08	0.01
588	0.055	0.054	0.068	0.06	0.00

(Equation 1) Fv/Fm = (Fm − Fo)/Fm

• In Excel, calculate the average quantum yield of the triplicates using the function *AVERAGE.* Calculate the standard error using the function *STDEV(range)/SQRT(3).*
• Plot the effective QY of PSII versus the photosynthetically active radiation (PAR, μmol m^−2^ s^−1^) and compare light stress response.

To identify DEGs in the various samples during surface colonization, use the DESeq2 R package (Love et al., 2014). Using the negative binomial distribution, the DESeq2 method provides a differential analysis of RNA-seq count data. The package and reference manual are available at http://www.bioconductor.org/packages/release/bioc/html/DESeq2.html.

• To reduce the memory size of the data object, pre-filter each sample by removing the raw read counts <10.
• Using the single *DESeq* function implemented in DESeq2, perform differential gene expression analysis after normalizing the total read count. Note that the *estimateSizeFactors* function occurs within the *DESeq* function. By default, the *geoMeans* argument takes the geometric mean of the read counts across all samples, thereby scaling the read counts by a reference sample.
• Use the *results* function to generate a table of the log2 fold changes and p-values.
• The *plotMA* function implemented in DESeq2 can be used to visualize the log2 fold changes against the mean of normalized counts.

Unless otherwise stated, a Student t-test should be performed to assess the statistical significance of physiological differences between samples. A p-value of less than 0.05 is used to reject the null hypothesis for comparisons between two groups.

## Limitations

While this protocol aims to identify key genes regulating the surface colonization process in the diatom *P. tricornutum*, it is possible that some regulatory genes are still not identified due to genome annotation limitations as well as the relatively low expression levels of some genes. Additionally, it is important to note that this protocol is primarily developed to manipulate the morphology shift of the model diatom *P. tricornutum* and the related surface colonization process. Therefore, we recommend following up this versatile protocol with an optimization workflow involving verifications of genome annotations as well as diatom RNA extraction methods to enable the successful application to other diatom species.

## Troubleshooting

### Problem 1

Low RNA yield and RNA degradation (referring to the step: Transcriptome analysis of two different morphotype cultures).

### Potential solution

When working with RNA instruments, DNase and RNase-free materials should be used, including pipette tips, tubes, and corresponding solutions. The work area/bench should be cleaned with an RNase inactivating solution before starting with the RNA extraction.

RNase activity is reduced with temperature; thus, unless otherwise stated, work with samples on ice or at 4°C at all times, and set the needed devices/centrifuges at 4°C.

### Problem 2

Low transformation efficiency (referring to the step: Transformation and selection of targeted morphotype strains).

### Potential solution

It is crucial that the electroporation is performed on the diatom cells at their exponential growth phase OD_700_ =0.2–0.4, 5.9 × 10^6^ cells (detailed protocol). A slight modification was introduced to the protocol (Fu et al., 2017), adding a washing step and making sure the cells are active before the electroporation.

Ensure using good quality purified DNA. It is recommended to store plasmids at −20°C for longer periods and minimize freeze-thaw cycles to avoid DNA degradation in the longer-term. Linearize the plasmid with the corresponding restriction enzymes prior to the electroporation. A validation step can be added before the transformation, consisting of running gel electrophoresis for the obtained linear fragment in order to validate the expected fragment size.

### Problem 3

Integration (non-targeted integration) of the reporter genes in the diatom genome (referring to the step: Transformation and selection of targeted morphotype strains).

### Potential solution

To examine the integration of the genes in the *P. tricornutum*, it is essential that the transformed cells (colonies) are sub-cultured 2–3 times in media with 100 μg/mL of zeocin for antibiotic resistance selection. After isolating genomic DNA from transformed cells, PCR verification is conducted on the extracted DNA to detect the inserted fragment using specific primers and validate the cDNA integration in the diatom genome.

### Problem 4

Specificity related to oval/fusiform cells (referring to the step: Preparing and cultivating two different morphotype cultures).

### Potential solution

Use microscopy to ensure that majority of the cells (>60%) of required form for corresponding analyses (oval *vs* fusiform)

To analyze the transcription shifts between solid and liquid cultures, RNA sequencing is performed on RNA isolated from the wild type *P. tricornutum* strain grown in liquid and on solid media. Prior to RNA extraction, cells should be observed under a bright-field microscope to ensure that the expected fusiform and oval morphotypes are the dominant morphotypes in liquid and solid cultures, respectively.

### Problem 5

Slow growth of *P. tricornutum* after transformation on zeocin-selective solid plates (referring to the step: Transformation and selection of targeted morphotype strains).

### Potential solution

To obtain single colonies of transformants on zeocin-selective solid plates as expected, it’s important to see diatom growth on plates within two weeks. The plates should be placed under relatively high humid (appropriately 80%) conditions to avoid drying out if cultivation duration is beyond two weeks. Alternatively, 1 mL of sterile f/2+Si medium could be added to the plates per week.

## Resource availability

### Lead contact

Further information and requests for resources and reagents should be directed to and will be fulfilled by the lead contact, Weiqi Fu (weiqifu@zju.edu.cn).

### Materials availability

All sequences of synthesized genes can be found in a recent publication (Fu et al., 2020).

## Data Availability

RNA-seq data from this article can be found in the GenBank/NCBI data libraries (GenBank: PRJNA566271). The RNA-seq data have also been deposited in Dryad with a unique identifier (https://doi.org/10.5061/dryad.ns1rn8ppx).
